# Introduction of Universal Newborn Screening for Sickle Cell Disease in Germany—A Brief Narrative Review

**DOI:** 10.3390/ijns7010007

**Published:** 2021-01-28

**Authors:** Stephan Lobitz, Joachim B. Kunz, Holger Cario, Dani Hakimeh, Andrea Jarisch, Andreas E. Kulozik, Lena Oevermann, Regine Grosse

**Affiliations:** 1 Gemeinschaftsklinikum Mittelrhein, Pädiatrische Hämatologie und Onkologie, Koblenzer Straße 115-155, 56073 Koblenz, Germany; 2 Department of Pediatric Oncology, Hematology and Immunology, Hopp-Children’s Cancer Center (KiTZ) Heidelberg, University of Heidelberg, Im Neuenheimer Feld 430, 69120 Heidelberg, Germany; joachim.kunz@med.uni-heidelberg.de (J.B.K.); andreas.kulozik@med.uni-heidelberg.de (A.E.K.); 3 Universitätsklinikum Ulm, Klinik für Kinder- und Jugendmedizin, Pädiatrische Hämatologie und Onkologie, Eythstrasse 24, 89075 Ulm, Germany; holger.cario@uniklinik-ulm.de; 4 Campus Virchow-Klinikum, Charité-Universitätsmedizin Berlin, Klinik für Pädiatrie m.S. Onkologie/Hämatologie/SZT, Augustenburger Platz 1, 13353 Berlin, Germany; dani.hakimeh@charite.de (D.H.); lena.oevermann@charite.de (L.O.); 5 Zentrum für Kinder- und Jugendmedizin, Klinikum der Johann-Wolfgang-Goethe-Universität, Schwerpunkt Stammzelltransplantation und Immunologie, Theodor-Stern-Kai 7, 60590 Frankfurt am Main, Germany; a.jarisch@kgu.de; 6 Zentrum für Geburtshilfe, Kinder- und Jugendmedizin, Universitätsklinikum Eppendorf, Klinik und Poliklinik für Pädiatrische Hämatologie und Onkologie, Martinistr. 52, 20246 Hamburg, Germany; r.grosse@uke.de

Sickle cell disease (SCD) is a severe non-malignant disorder of hemoglobin and is inherited in an autosomal-recessive manner. SCD is most prevalent in areas where malaria is endemic or has been endemic in the past, like Sub-Saharan Africa, the Middle East and India. However, because of voluntary migration and forced displacement, it has also become more prevalent in Central and Northern European countries within the last two decades. Some countries have already implemented newborn screening (NBS) programs for SCD in the past (Table 1). On 20 November 2020, the Federal Joint Committee (“Gemeinsamer Bundesausschuss”, G-BA), the top self-governing body in the German healthcare system has decided to introduce NBS for SCD in Germany [1].

The German example shows that the implementation of NBS for SCD is also possible in Central and Northern European countries with comparatively low prevalence. However, the highly political process requires an accurate preparation with a focus on the generation of robust epidemiological data and a strategy to convince authorities that affected newborns will benefit from its early identification. While the screening procedure itself may be technically trivial, it needs to be embedded into a disease management program. As a minimum, it is mandatory to ensure provision of state-of-the-art care for patients identified by NBS (e.g., by adhering to established treatment guidelines) and to follow each individual’s clinical course by means of a patient registry.

The G-BA decision was the fortunate endpoint of a one-decade process which began at the occasion of the second Pan-European Conference on Haemoglobinopathies in Berlin in March 2010. This conference organized by the Thalassemia International Federation (TIF) was held in Berlin to emphasize the increasing prevalence of haemoglobinopathies in Central and Northern Europe. During the conference, the lack of prevalence data on SCD in Germany became obvious. Therefore, the idea arose to generate robust epidemiological figures by a universal NBS pilot study with the ultimate goal of introducing routine NBS for SCD in Germany as the most important measure to make an early diagnosis.

It took a lot of persuading to gain funding and ethics approval for NBS pilot studies since haemoglobinopathies were generally not considered an emerging healthcare problem by the beginning of the last decade in Germany. Finally, the first study was supported by a pharmaceutical company and through the provision of preferential prices for screening hardware and consumables. It was conducted in Berlin (Berlin I study) and strikingly demonstrated the presence of an unexpectedly high birth prevalence of SCD (1:2435) and, thus, the need for more epidemiological data from both presumed high- and low prevalence areas [4,5]. The results of Berlin I paved the way for three additional studies soon thereafter.

The Hamburg study exactly recapitulated the Berlin results (1:2385). As expected, the Heidelberg study showed a lower birth prevalence (1:12,613) in the mixed rural-metropolitan catchment area of the Heidelberg screening laboratory in Southwest Germany [6,7]. A follow-up project in Berlin (Berlin II study) was expanded to the rural Brandenburg region surrounding the German capital. The birth prevalence of children with SCD was significantly lower in this study (1:4154) than in Berlin I, but it underlined the highly relevant prevalence of SCD in this region [8]. Based on these four studies (Table 2) and a supportive publication analyzing health insurance data [9], a birth prevalence of 1:5000–1:7500 was estimated for the whole of Germany, corresponding to 100–160 newborns with SCD per year assuming an annual number of births of 800,000.

Interestingly, four different methodological approaches were used in the studies (Berlin I: 1st tier HPLC/2nd tier CE; Hamburg: HPLC/allele-specific hybridization assay; Heidelberg: TaqMan assay/Sanger sequencing; Berlin II: MS/MS, CE) (Table 2). All proved to be easy to perform and highly reliable and had a similar magnitude of costs per sample. The Berlin II study was awarded the “Eva Luise Köhler Prize”, an award offered by the scientific foundation of Germany’s former presidential wife for its MS/MS approach.

In parallel, the preparations for a comprehensive disease management program started. After a peer-reviewed competitive application process, the General Assembly of the German Society for Pediatric Oncology and Hematology (GPOH) mandated a group of pediatricians and scientists, the GPOH-Konsortium Sichelzellkrankheit, to take over the responsibility for this program in 2012.

The consortium developed and published a comprehensive treatment guideline (https://www.awmf.org/leitlinien/detail/ll/025-016.html). In parallel, a web-based patient registry (funded by the Deutsche Kinderkrebsstiftung) was implemented. Two laboratories started to offer reference diagnostics for primary and secondary disease modifiers (*HBB* and *HBA1/2* genotype, various genetic determinants of HbF persistence). These pillars of the program were supplemented by an online information portal, educational articles for physicians in German-language journals, a newly established three-day annual conference on non-malignant blood diseases, continuous presentations at various national conferences and a modular educational program for patients and their families. The group developed a smartphone app, a patient passport with an emergency card and a booklet for patients. This “sickle cell companion” gives a concise overview about the recommended routine blood tests and clinical examinations including transcranial Doppler examinations, echocardiography, etc. The consortium also organizes case conferences to facilitate decision making. Finally, scientific projects were initiated, and the first data have recently been published [10,11].

The proposal to introduce a universal NBS for SCD in Germany was submitted to the G-BA in March 2018. The application was reviewed with regards to feasibility, risks and benefits and economic viability. The process was complemented by an independent literature search, expert hearings and two surveys among relevant medical and scientific societies. The decision benefitted from a European consensus statement and three epidemiological papers published in the crucial phase [2,9,10,11]. One paper showed a significant increase of hospitalizations for SCD complications from a statistical analysis of health insurance data for the years 2007–2015 [11]. Another work that was based on data from the largest health insurance provider in Germany demonstrated evidence of a significant number of children born with SCD in 2009 and 2010 [9]. The author used an indirect approach since there is no statistical recording of non-malignant blood diseases in Germany. In addition, the first paper from the newly established German SCD registry strikingly demonstrated that 80% of SCD patients in Germany were diagnosed not only too late, but also after significant complications had already occurred [10]. These publications were clearly in favor of a positive decision on the NBS application [2,9,10,11]. The last step was to obtain a positive vote of the German genetic diagnostics commission (GEKO) at the Robert Koch Institute (RKI), an institution of the German Federal Ministry of Health. The RKI vote was required because NBS for SCD falls under the very strict German Genetic Testing Act that prohibits making a genetic diagnosis in minors that are not of direct medical relevance. These regulations do not allow the identification of sickle cell carriers by NBS. Finally, the G-BA consented.

The next steps will now be to define a list of specialized hematology services, mostly in tertiary care institutions, where screening-positive newborns will be referred to not only for diagnostic confirmation of the screening result but also for the initiation of long-term clinical care. To evaluate the benefit of the newly introduced NBS for SCD in the context of the powerful German healthcare system, it will be of utmost importance that the majority of patients will be enrolled in the national SCD registry and treated according to the current national treatment guideline. Chances are that in three to five years, we will know if affected individuals will benefit from NBS for SCD in Germany.

## Figures and Tables

**Table 1:** NBS programs for SCD in Europe.

Country	Level	Coverage	Birth Prevalence
Belgium	regional * (Brussels & Liège)	universal	1:2300
France	national	targeted in metropolitan France and universal in overseas territories	1:1800
Germany	national	universal	1:5000–1:7500 (est.)
Ireland	national *	targeted	10–15 cases p.a.
Malta	national	universal	no case in 2017
Netherlands	national	universal	1:5800
Spain	national	universal	1:8300
United Kingdom	national	universal	1:2200

Adapted from reference [2,3]; * no public funding.

**Table 2:** Overview about the four German pilot studies on NBS for SCD.

	Berlin I	Hamburg	Heidelberg	Berlin II
**mode**	universal	universal	universal	universal
***n***	34,084	17,018	37,838	29,079
**first tier method**	HPLC	HPLC	TaqMan assay	MS/MS
**second tier method**	CE	Hybridization assay	Sanger sequencing	CE
**carriers**	165	98	91	134
**diagnosed**	14	8	3	7
**genotypes**	9 × SCD-S/S 1 × SCD-S/β thal. 4 × SCD-S/C	3 × SCD-S/S 5 × SCD-S/C	3 × SCD-S/β thal.	4 × SCD-S/S 2 × SCD-S/C 1 × SCD-S/HPFH
**calculated local birth prevalence**	1:2435	1:2127	1:12,613	1:4154

## Abbreviations

CE	capillary electrophoresis
HPFH	hereditary persistence of fetal hemoglobin
HPLC	high performance liquid chromatography
MS/MS	tandem mass spectrometry
